# A start codon-targeted genome editing strategy for generating hypomorphic mutants of lethal plant genes

**DOI:** 10.5511/plantbiotechnology.25.0812a

**Published:** 2025-12-25

**Authors:** Mika Yoshimura, Tsubasa Mamiya, Naoki Takahashi, Takashi Ishida

**Affiliations:** 1 Faculty of Advanced Science and Technology, Kumamoto University, 2-39-1 Kurokami, Kumamoto, Kumamoto 860-8555, Japan; 2 Department of Life Sciences, School of Agriculture, Meiji University, 1-1-1 Higashi-mita, Tama-ku, Kawasaki, Kanagawa 214-8571, Japan

**Keywords:** CRISPR-Cas9, DNA damage response, genome editing, hypomorphic mutant allele, NSE1

## Abstract

In plants, the functional characterization of essential genes is often hindered by the lethality associated with complete loss-of-function alleles. Here, we present a genome editing-based strategy to generate viable hypomorphic alleles through selective removal of the translation start codon. Using *Arabidopsis thaliana* *NON-SMC ELEMENT 1*, which encodes a conserved component of the Structural Maintenance of Chromosomes (SMC) 5/6 complex involved in DNA repair and genome stability, as a model, we generated CRISPR-Cas9-edited alleles lacking the start codon. These homozygous mutants exhibited severe developmental defects, including stunted growth and failure to form true leaves. Moreover, they displayed molecular hallmarks of genome instability, such as increased DNA fragmentation, upregulation of DNA repair and cell cycle checkpoint genes, and root meristem cell death. Complementation assays using wild-type and mutated *NSE1* genomic constructs confirmed that these alleles retained partial gene function. Overall, the use of start codon removal as an editing strategy is a robust and broadly applicable approach for generating hypomorphic alleles without relying on transcript-level manipulations, such as RNAi. This work therefore provides a practical demonstration of a novel editing strategy for dissecting the functions of essential genes that are otherwise genetically intractable. Consequently, this approach expands the functional genomics toolkit and opens new avenues for basic plant biology and advanced biotechnological applications.

Functional analyses of specific genes in plant science require meticulous genetic analysis. Bioresources, such as a collection of mutants, have been used to accelerate our understanding of gene function, and recent advances in genome editing have revolutionized multiple fields in the life sciences. However, many mutants largely eliminate the function of the gene of interest, which makes them unsuitable for studying genes where complete loss-of-function is lethal. To overcome this difficulty, gene knockdown technologies such as RNAi are often used to elucidate gene function. Recently, we developed a method for creating viable mutant alleles by using CRISPR-Cas9 to introduce mutations into a splicing acceptor site (Yoshimura and Ishida 2025). However, since the applicability of this strategy may vary depending on gene structure, we now propose a new methodology that makes hypomorphic allele generation more versatile through genome editing, thereby stimulating alternative approaches for producing diverse mutants.

In any given gene, if the primary start codon functions incorrectly during translation, initiation from an alternative start codon may produce a protein that is incomplete and retains partial functionality (Makino et al. 2016). Based on this observation, we hypothesized that the removal of a start codon via genome editing can generate hypomorphic alleles. To test this possibility, we targeted the *Arabidopsis thaliana NON-SMC ELEMENT 1* (*NSE1*) gene. The protein encoded by *NSE1*, which was originally identified via its yeast homolog, is a component of the conserved eukaryotic SMC5/6 complex (Fujioka et al. 2002). This complex is known to play a crucial role in DNA repair and maintaining genome stability (Aragón 2018; Diaz and Pecinka 2018). In plants, the critical importance of the Structural Maintenance of Chromosomes (SMC) 5/6 complex is well documented. For example, loss-of-function mutations in its components generally result in embryonic lethality or, if embryos are viable, severe developmental defects (Diaz and Pecinka 2018; Ishida et al. 2009; Yang and Pecinka 2022). Moreover, two T-DNA insertion alleles in the intron of *NSE1* have been reported, and both have been described as embryonic lethal mutants (Figure 1A) (Li et al. 2017, 2019). Consequently, analyses of *NSE1* have largely been confined to studies of embryogenesis, with post-germination functions assessed using transgenic plants in which ectopic expression from non-native promoters is used to partially rescue the mutant phenotype. Given this context, we posited that generating hypomorphic alleles of *NSE1* could substantially aid in its functional dissection.

**Figure 1:**
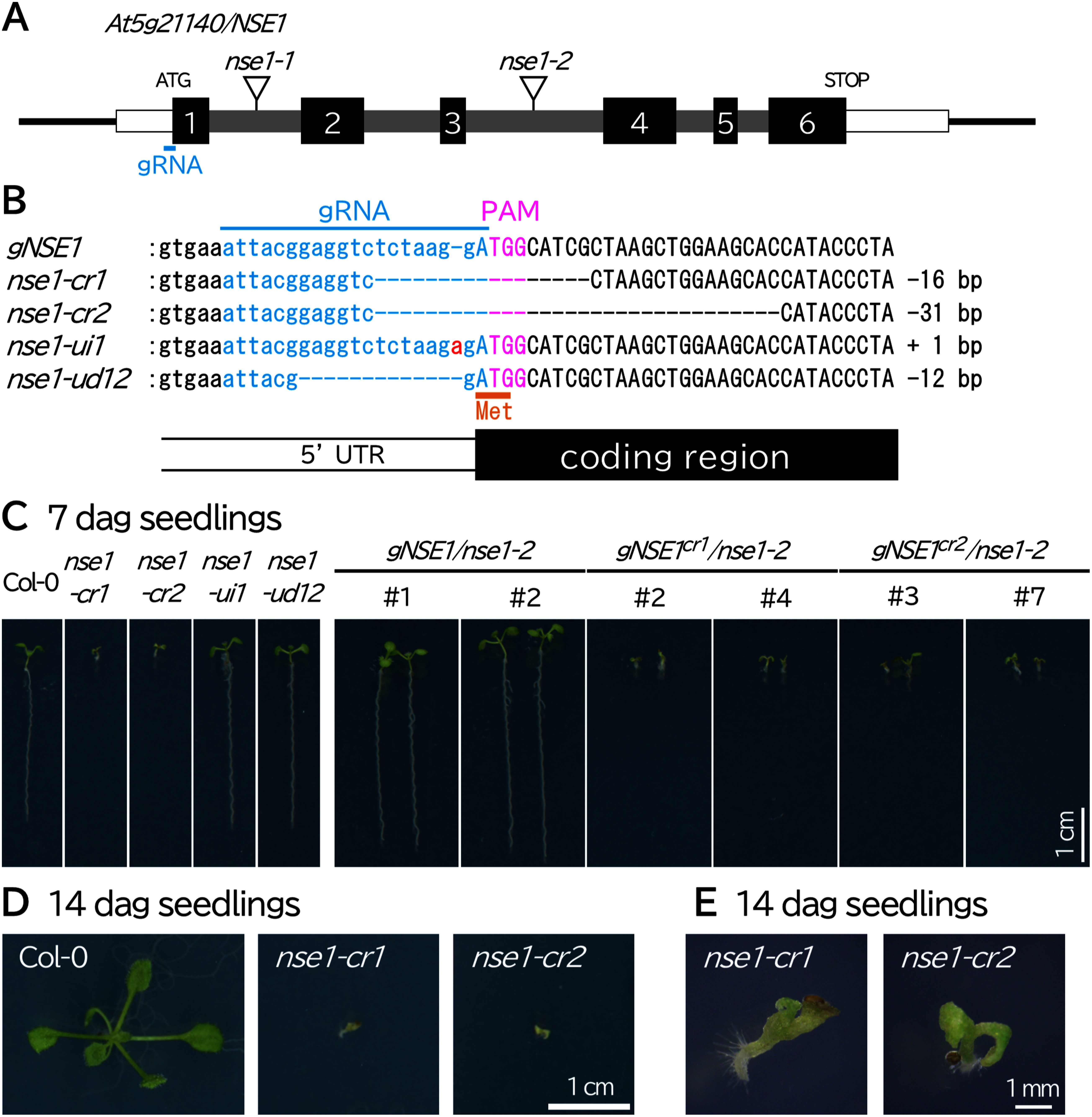
Figure 1. CRISPR-generated *nse1* mutants and their developmental phenotypes. (A) Structure of the *At5g21140*/*NSE1* gene. Black boxes indicate *NSE1* coding regions, gray boxes indicate introns, and white boxes indicate UTRs. The positions of *nse1-1* (CS16151) and *nse1-2* (CS24066) T-DNAs are indicated by triangles. The sky-blue bar indicates the gRNA position and the protospacer-adjacent motif (PAM). (B) Genomic sequences of wild-type (Col-0) and CRISPR-Cas9-edited mutant alleles. Pink letters indicate PAMs, sky-blue letters indicate gRNAs predicted to be responsible for the mutations, and red letters indicate an inserted nucleotide. Orange letters and an orange bar indicate the position of the translation initiation codon (ATG). The position of the 5′UTR-first exon junction is shown below the genomic DNA alignment. (C) Seven-day-old seedlings of wild-type (Col-0), *nse1-cr1*, *nse1-cr2*, *nse1-ui1*, *nse1-ud12*, *gNSE1/nse1-2 #1*, *gNSE1/nse1-2 #2*, *gNSE1^cr1^/nse1-2 #2*, *gNSE1^cr1^/nse1-2 #4*, *gNSE1^cr2^/nse1-2 #3*, and *gNSE1^cr2^/nse1-2 #7*. (D) Fourteen-day-old seedlings of the wild-type (Col-0), *nse1-cr1*, and *nse1-cr2*. (E) Dissection micrographs of 14-day-old seedlings of the *nse1-cr1* and *nse1-cr2* mutants. Scale bar=1 cm in (C) and (D), 1 mm in (E).

Accordingly, we used the CRISPRdirect online tool to design guide RNAs capable of inducing cleavage immediately upstream of the translation initiation codon. Subsequently, we constructed genome editing vectors employing the pDe-Cas9 system using a previously described method (Fauser et al. 2014; Naito et al. 2015; Yamaguchi et al. 2017; Yamamoto et al. 2019). Next, in the T_2_ generation, we isolated mutants harboring either a 16-bp or a 31-bp deletion in one allele; these were designated as *nse1-cr1* and *nse1-cr2*, respectively (Figure 1B, Supplementary Figure S1). Both alleles likely lack the first coding region that contains the start codon. Intriguingly, we recovered homozygous seedlings from heterozygote parents of *nse1-cr1* and *nse1-cr2*, indicating that these mutant alleles remain viable, at least during embryogenesis (Figure 1C). Thus, in contrast to the previously reported T-DNA insertion alleles, which are embryonically lethal, these mutants were classified as hypomorphic alleles.

We further characterized the morphology of the *nse1-cr1* and *nse1-cr2* mutants. To this end, mutant seedlings were grown for seven days on half-strength MS agar medium; they were found to be markedly dwarfed relative to wild-type seedlings (Figure 1C). Moreover, at 14 days after germination, most mutants failed to develop true leaves, and their roots were so reduced that they were barely visible under a dissecting microscope (Figure 1D, E). Finally, none survived to the reproductive stage. Next, we took advantage of the availability of viable homozygous mutant seedlings and investigated the biological relevance of *NSE1*. Using comet assays to quantify DNA integrity loss, we found that *nse1-cr1* and *nse1-cr2* mutants exhibited pronounced DNA fragmentation (Figure 2A, B). Furthermore, the expression levels of the DNA repair genes *BRCA1*, *RAD17*, and *PARP2*, which are induced by DNA damage, were strongly elevated in both mutants (Figure 2C) (Ogita et al. 2018). Similarly, *SMR5* and *SMR7*, which are two important regulators of the cell cycle checkpoint and are expressed in response to DNA damage, were strongly upregulated in mutant seedlings (Figure 2C) (Yi et al. 2014). As part of the DNA damage response, we also observed cell death in the root apical meristem (Figure 2D). Overall, these findings are consistent with those of previous studies employing an ectopic *NSE1* expression line and further highlighting the usefulness of our strategy for enabling the recovery of viable hypomorphic mutants for functional analysis.

**Figure 2:**
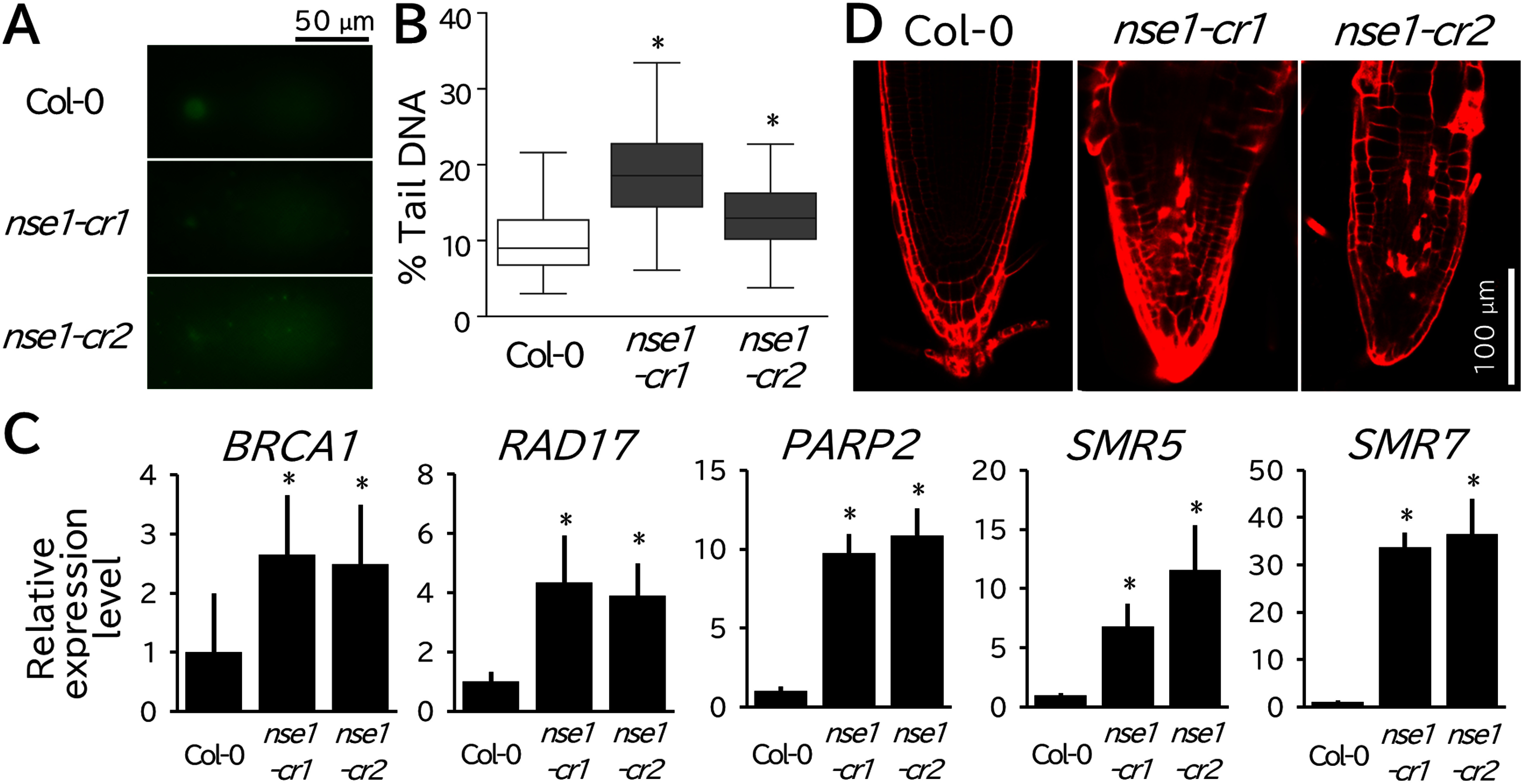
Figure 2. Genome stability is impaired in hypomorphic *nse1* mutants. (A) An alkaline comet assay was performed on seven-day-old wild-type (Col-0), *nse1-cr1*, and *nse1-cr2* plants using a Comet Assay Single Cell Gel Electrophoresis Assay (R&D Systems, Inc.). (B) Percentage of DNA content in the tails of independent nuclei in wild-type (Col-0), *nse1-cr1*, and *nse1-cr2* plants. Box plots were generated from 200, 199, and 143 individual measurements, respectively. Asterisks indicate significant differences (Mann–Whitney *U* test, *p*<0.05). (C) Relative expression levels of *BRCA1*, *RAD17*, *PARP2*, *SMR5*, and *SMR7*. All expression levels were normalized to the levels of *ACTIN2*. Graphs indicate mean±SD as calculated using three biological replicates. Asterisks indicate significant differences (Student’s *t* test, *p*<0.05). All primers used in this assay are shown in Supplementary Table S1. (D) Confocal microscopy images of PI-stained roots from seven-day-old wild-type (Col-0), *nse1-cr1* and *nse1-cr2* plants. Scale bar=50 µm in (A), 100 µm in (D).

In parallel, we isolated additional genome-edited alleles. One, *nse1-ui1*, carried a 1-bp insertion, and another, *nse1-ud12*, carried a 12-bp deletion; both mutations were located in their respective 5′ UTR regions (Figure 1B, Supplementary Figure S1). Interestingly, homozygous plants harboring these alleles exhibited phenotypes that were nearly indistinguishable from the wild-type, thereby reinforcing the notion that loss of the start codon is critical for generating hypomorphic alleles (Figure 1C).

Notably, the *NSE1* sequences of *nse1-cr1* and *nse1-cr2* exhibited residual functional capacity, indicating that they are not nonfunctional. To further examine these functions, we conducted a complementation assay using a genomic fragment of *NSE1*. Specifically, we cloned a 4,646-bp fragment and introduced it into a *nse1-2* mutant. This fully restored normal seedling development confirms that the fragment was sufficient to restore *NSE1* function (Figure 1C). We then engineered modified genomic sequences with deletions analogous to those found in the *nse1-cr1* and *nse1-cr2* mutants by site-directed mutagenesis. When these constructs were transformed into the *nse1-2* mutant, they reproduced a dwarf phenotype reminiscent of that observed in the original hypomorphic alleles (Figure 1C). Taken together, these findings provide genetic evidence that the *nse1-cr1* and *nse1-cr2* alleles compromise but do not eliminate *NSE1* function, consistent with their classification as hypomorphic.

In conclusion, this study demonstrates that targeted removal of a start codon via genome editing is an effective and broadly applicable strategy for generating hypomorphic alleles in *Arabidopsis*. By directly disrupting translation initiation, this approach enables the generation of viable mutant alleles even in genes for which complete loss-of-function is embryonically lethal—an outcome often unattainable with conventional knockout or RNAi-based methods. On the other hand, a key limitation of this strategy lies in the uncertainty regarding the exact transcripts and protein products generated by the mutant alleles. Nonetheless, the method offers a significant advantage in its high degree of design flexibility. Similar outcomes can likely be achieved using other gene targeting technologies (e.g., TALENs) or DNA rewriting strategies, such as base editing or prime editing. Applying this approach to NSE1, a component of the SMC5/6 complex, we successfully recovered viable hypomorphic mutants that displayed characteristic defects in development and genomic integrity. These findings lay the groundwork for future investigations into the genetic interplay between NSE1—as a component of the SMC5/6 complex—and canonical DNA damage regulators. In particular, studies focusing on ATM and ATR, which are key kinases that sense DNA damage and initiate signaling cascades, and SOG1, a master regulator of the DNA damage response, will be important for understanding the broader regulatory network. Furthermore, our results may guide investigations into the molecular mechanisms underlying cell cycle checkpoint control mediated by SMR and related factors. Overall, our work expands the toolkit for functional gene analysis and offers a promising avenue for basic research and biotechnological applications in the future.

## Abbreviations

CRISPR-Cas9: clustered regularly interspaced short palindromic repeats/CRISPR-associated proteins 9
NSE1: non-SMC element 1
SMC: structural maintenance of chromosomes

## Description of supplementary files

Supplementary Figure S1. Schematic representation of mutant isolation processes.

Supplementary Table S1. Primers used in this study.
