# Supplementary material for: A start codon-targeted genome editing strategy for generating hypomorphic mutants of lethal plant genes
Source: Plant Biotechnol (Tokyo). 2025 Dec 25;42(4):509–12. doi: 10.5511/plantbiotechnology.25.0812a (PMC12781906; doi:10.5511/plantbiotechnology.25.0812a)
Supplement: Supplementary Data [file plantbiotechnology-42-4-25.0812a-s001.pdf]

Figure S1

T0 Agrobacterium-mediated transformation with 4 Col-0 (Wild-type) plants

T1 T1 selection:
1. Select transformants with glufosinate-ammonium
2. Evaluation of efficiency by PCR-RE assay (primer P1 and P2, DdeI)

14 T1 seedlings were selected

T2 Selection of T-DNA free (Cas9-free) seedlings to avoid extra mutagenesis.

105 T2 seedlings / 594 T2 seeds were selected

T2 line	glufosinate-ammonium sensitive	total seedlings
1	18	74
2	0	89
4	20	77
7	10	89
8	16	86
11	18	89
14	23	90

PCR assay for large deletion (primer P3 and P4)
nse1-cr1
nse1-cr2
nse1-ud12

PCR-RE assay for small In/Del (primer P1 and P2, DdeI)
nse1-ui1

Supplementary Figure S1 Schematic representation of the mutant isolation processes. The mutations were expected to be primarily induced in the T1 generation. We selected Cas9-free seedlings in the T2 generation, and then selected the mutants using a PCR assay or a PCR-RE assay. Subsequently, they were propagated to obtain homozygous or heterozygous mutant seeds in the T3 generation.

Supplementary Table S1 Primers used in this study

primer name	sequence
gRNA construction	
CR-NSE1-F	acggaggtctctaaggAGTTTTAGAGCTAGAAATAGCAAG
CR-NSE1-R	ccttagagacctccgtaatCAATCACTACTTCGACTCTA
pUC19-IF-M13F	ctcggtacccggggatcGTAAAACGACGGCCAGT
pUC19-IF-M13R	cgactctagaggatcGGAAACAGCTATGACCATG
Genotyping	
pBI_LBb1.3	ATTTTGCCGATTTCGGAAC
SALK_136483-RB	GCTGATGATCAATCCAAACTCGGG
SALK_136483-LB	GTTGACTGCTGCTGGCTTCACTTG
NSE1-dCAPS-DdeI-F (P1)	aataattttatggcgtcatcgtgtcgataacgcttcccgc
NSE1-dCAPS-DdeI-R (P2)	AATCAAAGCCTGAATTAGGGTATGGTGCTTCCAGgTTAGC
NSE1-delF (P3)	ttcacagaaacaatcagggttcacc
NSE1-delR (P4)	GAAATTCTTTCTCCTTGAGAGGACC
genomic fragments cloning/ construction/ sequencing	
TOPO-NotI-IF-gNSE1-F	AAAAGCAGGCTCCgcggccgcgatgcatcgatgtggctacatgacc
TOPO-AscI-IF-gNSE1-R	AGAAAGCTGGGTCggcgcgcctagttttgtttatgccttgatgggc
NSE1-M1F	tctcagtttgaccgtttgac
NSE1-M2F	aaacggtccgcaaatgatcc
NSE1-M3F	acggaattaccacgcctcttaacc
NSE1-M4F	tactgggtaccttgatgttg
NSE1-M5F	CGATTGGAGAATCAGgtcag
NSE1-M6F	TTCTTGACCTTCGCAGTTGG
NSE1-M7F	aagaactccggttttagggc
gNSE1-mutR	gacctccgtaatttcacagtatg
gNSE1-mutF1	gaaattacggaggtcCTAAGCTGGAAGCACCATAC
gNSE1-mutF2	gaaattacggaggtcCATACCCTAATTCAGGCTTTG
RT-qPCR	
BRCA1-F	AAAGCCCAATCCAGGTGACA
BRCA1-R	CTAGGACCTTGGCCTTCACAG
RAD17-F	AGGATGTTCCACAGTCAGCC
RAD17-R	CGAATCACGTGCCATCTGGA
PARP2-F	ATGCTACTCTGGCACGGTTCAC
PARP2-R	AGGAGGAGCTATTCGCAGACCTTG
SMR5-F	AACTACGACGACGGAGATACG
SMR5-R	GCAACTAGGTTGCCGCTTG
SMR7-F	GCCAAAACATCGATTCGGGC
SMR7-R	CTCCGGAGTCTTTGCTCCTC
ACT2-F	CTGGATCGGTGGTTCCATTC
ACT2-R	CCTGGACCTGCCTCATCATAC
